# Development of a diagnostic multivariable prediction model of a positive SARS-CoV-2 RT-PCR result in healthcare workers with suspected SARS-CoV-2 infection in hospital settings

**DOI:** 10.1371/journal.pone.0316207

**Published:** 2024-12-26

**Authors:** Sandra Liliana Valderrama-Beltrán, Juliana Cuervo-Rojas, Martín Rondón, Juan Sebastián Montealegre-Diaz, Juan David Vera, Samuel Martinez-Vernaza, Alejandra Bonilla, Camilo Molineros, Viviana Fierro, Atilio Moreno, Leidy Villalobos, Beatriz Ariza, Carlos Álvarez-Moreno

**Affiliations:** 1 Faculty of Medicine, Department of Clinical Epidemiology and Biostatistics, PhD Program in Clinical Epidemiology, Pontificia Universidad Javeriana, Bogotá, Colombia; 2 Faculty of Medicine, Department of Internal Medicine, Division of Infectious Diseases, Pontificia Universidad Javeriana, Hospital Universitario San Ignacio, Infectious Diseases Research Group, Bogotá, Colombia; 3 Faculty of Medicine, Department of Clinical Epidemiology and Biostatistics, Pontificia Universidad Javeriana, Bogotá, Colombia; 4 Faculty of Medicine, Pontificia Universidad Javeriana, Hospital Universitario San Ignacio, Bogotá, Colombia; 5 Human Resources Office, Hospital Universitario San Ignacio, Bogotá, Colombia; 6 Faculty of Medicine, Department of Internal Medicine, Division of Emergency, Pontificia Universidad Javeriana, Hospital Universitario San Ignacio, Bogotá, Colombia; 7 Clinical Laboratory, Clinical Laboratory Science Research Group, Hospital Universitario San Ignacio, Bogotá, Colombia; 8 Clínica Colsanitas and Facultad de Medicina, Universidad Nacional de Colombia, Bogotá, Colombia; Norbert Wiener University, PERU

## Abstract

**Background:**

Despite declining COVID-19 incidence, healthcare workers (HCWs) still face an elevated risk of Severe Acute Respiratory Syndrome Coronavirus 2 (SARS-CoV-2) infection. We developed a diagnostic multivariate model to predict positive reverse transcription polymerase chain reaction (RT-PCR) results in HCWs with suspected SARS-CoV-2 infection.

**Methods:**

We conducted a cross-sectional study on episodes involving suspected SARS-CoV-2 symptoms or close contact among HCWs in Bogotá, Colombia. Potential predictors were chosen based on clinical relevance, expert knowledge, and literature review. Logistic regression was used, and the best model was selected by evaluating model fit with Akaike Information Criterion (AIC), deviance, and maximum likelihood.

**Results:**

The study included 2498 episodes occurring between March 6, 2020, to February 2, 2022. The selected variables were age, socioeconomic status, occupation, service, symptoms (fever, cough, fatigue/weakness, diarrhea, anosmia or dysgeusia), asthma, history of SARS-CoV-2, vaccination status, and population-level RT-PCR positivity. The model achieved an AUC of 0.79 (95% CI 0.77–0.81), with 93% specificity, 36% sensitivity, and satisfactory calibration.

**Conclusions:**

We present an innovative diagnostic prediction model that as a special feature includes a variable that represents SARS-CoV-2 epidemiological situation. Given its performance, we suggest using the model differently based on the level of viral circulation in the population. In low SARS-CoV-2 circulation periods, the model could serve as a replacement diagnostic test to classify HCWs as infected or not, potentially reducing the need for RT-PCR. Conversely, in high viral circulation periods, the model could be used as a triage test due to its high specificity. If the model predicts a high probability of a positive RT-PCR result, the HCW may be considered infected, and no further testing is performed. If the model indicates a low probability, the HCW should undergo a COVID-19 test. In resource-limited settings, this model can help prioritize testing and reduce expenses.

## Introduction

Severe Acute Respiratory Syndrome Coronavirus 2 (SARS-CoV-2) infection has led to millions of deaths globally. It is now an endemic public health problem worldwide. Consequently, the World Health Organization (WHO) and the Center for Disease Control and Prevention (CDC) continue to advocate vaccination against SARS-CoV-2 and the active search for cases in healthcare workers (HCWs) [1–3]. The introduction of vaccination, change in the epidemiological situation and limited healthcare resources led to prioritizing COVID-19 diagnosis for certain groups like HCWs, in several countries, including ours [4].

Frontline workers, especially those in high-risk units such as nurses and those in close contact with patients, are particularly vulnerable to infection due to occupational exposure [5–9]. Testing of HCWs plays a crucial role in reducing illness rates and minimizing the spread of SARS-CoV-2 within healthcare settings [10]. This prioritization is important for preventing potential cross-transmission of the infection between HCWs and high-risk patients, with transmission rates reported to be as high as 44% [11,12].

One possible strategy for SARS-CoV-2 diagnosis in HCWs involves using models that predict a positive RT-PCR result. RT-PCR is considered the most accurate method for identifying symptomatic and asymptomatic infection cases [13,14].

These models aim to rapidly classify suspected cases without immediate use of RT-PCR or other tests, potentially optimizing resources and reducing costs. However, most available prediction models are designed for the general population, not specifically for HCW [15,16]. Few studies include Latin American populations, and many rely only on data from initial epidemic waves, lacking information on the behavior of newer viral variants like Omicron, which is notably more transmissible and presents slightly different signs and symptoms, necessitating the development of updated predictive models. [17,18].

Hence, this study aimed to develop a statistical model specifically designed to predict a positive RT-PCR result for SARS-CoV-2 in HCWs with suspected SARS-CoV-2 infection.

## Methods

### Study design

A cross-sectional study was conducted on HCWs at Hospital Universitario San Ignacio (HUSI) in Bogotá, Colombia, who had signs and symptoms suggestive of COVID-19 or who had been in close contact with a person diagnosed with SARS-CoV-2 between March 6, 2020 and February 2, 2022. The study period covers the first four epidemic waves in Colombia [19]. The predominant variants during the period of the study were: ancestral, Gamma variant (P.1, P.1.x), Mu variant (B.1.621) and Omicron variant (BA.1.1), respectively [20].

### Setting

HUSI implemented a systematic monitoring program called "Linea Respira" to oversee HCWs who exhibited symptoms consistent with COVID-19 or had close contact with confirmed cases of SARS-CoV-2 infection. The program required prompt reporting of symptoms indicative of COVID-19, including the date of symptom onset, details of any close contact with confirmed cases, medical history, prior SARS-CoV-2 infection, and COVID-19 vaccination status, via email or a dedicated phone line to complete a web-based questionnaire. Then, they received a teleconsultation with a physician or were directed to the institutional emergency service based on symptom severity. Subsequently, they were scheduled for an RT-PCR test for SARS-CoV-2 within the following 72 hours, either at home or institutionally.

### Inclusion and exclusion criteria

The study included HCWs aged 18 years or older, working in inpatient and outpatient services of the HUSI. The unit of observation and analysis of this study were the episodes of symptoms or close contact of the HCWs in which an RT-PCR test was performed.

We excluded clinical episodes in which the RT-PCR test was taken after 10 days of symptom onset. Close contact episodes in which the RT-PCR test was taken within the first 48 hours of contact, or after fourteen days were excluded. To maintain independence between episodes, the following criteria were applied: If a HCW had a positive RT-PCR test and 15 days before had other negative RT-PCR test, only the positive result was considered to classify the episode. Additionally, episodes occurring within 90 days of an initial positive RT-PCR episode were not included. In cases where a HCW had multiple negative RT-PCR tests within 14 days, one of the episodes was selected by a random procedure.

### Identification of potential predictor variables

Based on existing literature, expert knowledge and availability of relevant data the following variables were preliminarily considered for prediction of a positive RT-PCR result for SARS-CoV-2 among HCWs with suspected infection: demographic factors such as age, sex assigned at birth, and socioeconomic status; occupational variables such as occupation, service, shift, and work in a COVID-19 designated area; general exposure and immunity indicators such as close contact with a case, history of COVID-19 and vaccination against SARS-CoV-2. We also considered known and potential clinical risk factors for infection such as underlying medical conditions (asthma, obesity, cardiovascular disease, immunosuppression), and reported symptoms including fever or chills, cough, dyspnea, rhinorrhea, odynophagia, anosmia or dysgeusia, headache, fatigue/weakness, myalgia or arthralgia, and diarrhea.

The set of variables also included the epidemiological situation of SARS-CoV-2 in Bogotá at the time of the episode. At the time of the study, reporting RT-PCR results was mandatory in the country and has remained so to the present. We considered that the percentage of positivity of the tests for SARS-CoV-2 performed in Bogotá could give appropriate information about the intensity of viral circulation in the population, which will define the risk of infection and consequently the probability of a positive result. For the model, for each episode we included the positivity in the city the day before the corresponding RT-PCR was done.

The outcome variable was the result of the RT-PCR for SARS-CoV-2 for the episode. RT-PCR was performed using nasopharyngeal swabs samples or aspirates collected using the VIASURE™ Real-Time PCR Detection Kit plates (CerTest BIOTEC, Zaragoza, Spain).

### Data collection

Data collection involved extracting information from the “Linea Respira” web-based questionnaire, occupational health surveillance databases, and clinical records of the episodes. The data were accessed for research purposes between June 15, 2022 and February 15, 2024. The authors had access to information that could identify individual participants during data collection, however, such identifying information was withdrawn after completing the date cleaning process. Data on positivity of tests in the city were obtained from the publicly available surveillance information provided by the health authorities of Bogotá and Colombia [21,22]. RT-PCR results were obtained from the HUSI clinical laboratory database and medical records. A researcher blind to the prediction variables collected the outcome variable.

### Sample size

To calculate the sample size required for model development we considered a maximum of 46 parameters to be included in the model. With an estimated RT-PCR positivity rate of 30% among HCWs, and aiming for at least 10 outcomes per parameter, we estimated the need of having at least 1,533 episodes of suspected SARS-CoV-2 infection in HCWs. In the end, our study included 2,498 episodes, having 27 outcomes for each of the 23 parameters included in the final selected model, ensuring an adequate sample size.

### Statistical analysis

Before analysis, we investigated outliers, potential data entry errors, and missing information. Data cleaning and completion of missing critical clinical information required the review of medical records, and in a few cases, contact with HCWs.

Missing data accounted for 4.1% of records, primarily in the work shift variable, presumed to be missing at random. Due to the low percentage of missing data and their nature, a complete-data analysis was chosen instead of imputation of missing values. There were no missing data for the outcome variable.

Continuous variables were described using medians and interquartile ranges (IQRs) according to the data distribution, while categorical variables were presented as absolute and relative frequencies.

The multivariable model was built using logistic regression. Initially, a complete model incorporated all predefined variables. Subsequently, a selection process identified the best model by considering clinically relevant variables and evaluating model fit using Akaike information criterion (AIC), deviance, and maximum likelihood ratio.

The linear relationship between the log odds of the outcome and the continuous variables was assessed with the “Box and Tidwell” test. We decided to apply a logarithmic transformation of age and RT-PCR positivity for SARS-CoV-2 to achieve model linearity. Model additivity and possible interactions between symptoms were evaluated, revealing a statistically significant interaction between fever and anosmia/dysgeusia, enhancing model fit and leading to its inclusion. There was no collinearity between the variables included in the model.

Thereafter, discrimination was evaluated via a receiver operating characteristic curve (ROC curve) and area under the ROC Curve (AUC) calculation with 95% confidence intervals. At the same time, calibration was assessed graphically and through the Hosmer and Lemeshow goodness-of-fit test. We also used the CORP reliability diagram approach, named by this acronym for its four properties: consistency, optimization, reproducibility, and group-adjacent violator (PAV)-based algorithm [23].

Furthermore, model sensitivity, specificity, and likelihood ratios were evaluated. Internal validation was performed using cross-validation to reassess model performance. In addition, another internal validation was carried out on the episodes during the fourth epidemic wave, dominated by the Omicron variant.

To further evaluate the clinical utility of the model considering the significance of the local epidemiological situation as indicated by the positivity of SARS-CoV-2 tests in the population as a crucial predictor of an individual’s RT-PCR result, we assessed how the model performs in scenarios of low and high SARS-CoV-2 circulation. Thus, the episodes were categorized into two groups: those occurring in periods with a population test positivity of 15% or higher (high viral circulation) and those occurring in periods with a positivity below 15% (low viral circulation) [24]. The original model with its parameters was then applied in each of these epidemiological scenarios. Subsequently, the model’s discrimination, calibration, and diagnostic performance were reassessed in these two scenarios.

Data analysis was conducted using R software version 4.1, using the packages blorr, UWbe536, pROC, generalhoslem, reliabilitydiag, and rms. This study followed theTRIPOD Statement and methodological recommendations for the development of prediction models [25,26].

### Research ethics considerations

This study was carried out following national (Resolution 8430 of 1993, of the Ministry of Health of Colombia) and International Standards (Declaration of Helsinki) for the ethical conduct of research. The study was approved by the Research and Ethics Committee of the HUSI and the School of Medicine of the Pontificia Universidad Javeriana (FM-CIEI-0686-21). We did not request consent from the participants given the retrospective nature of the study, which was based on clinical and laboratory records of an existing occupational health program of HUSI. Only four of the researchers had access to identified data during the process of data collection and cleaning, and then, we were careful to remove personal identifiers and assured the privacy of information.

## Results

**Fig 1:**
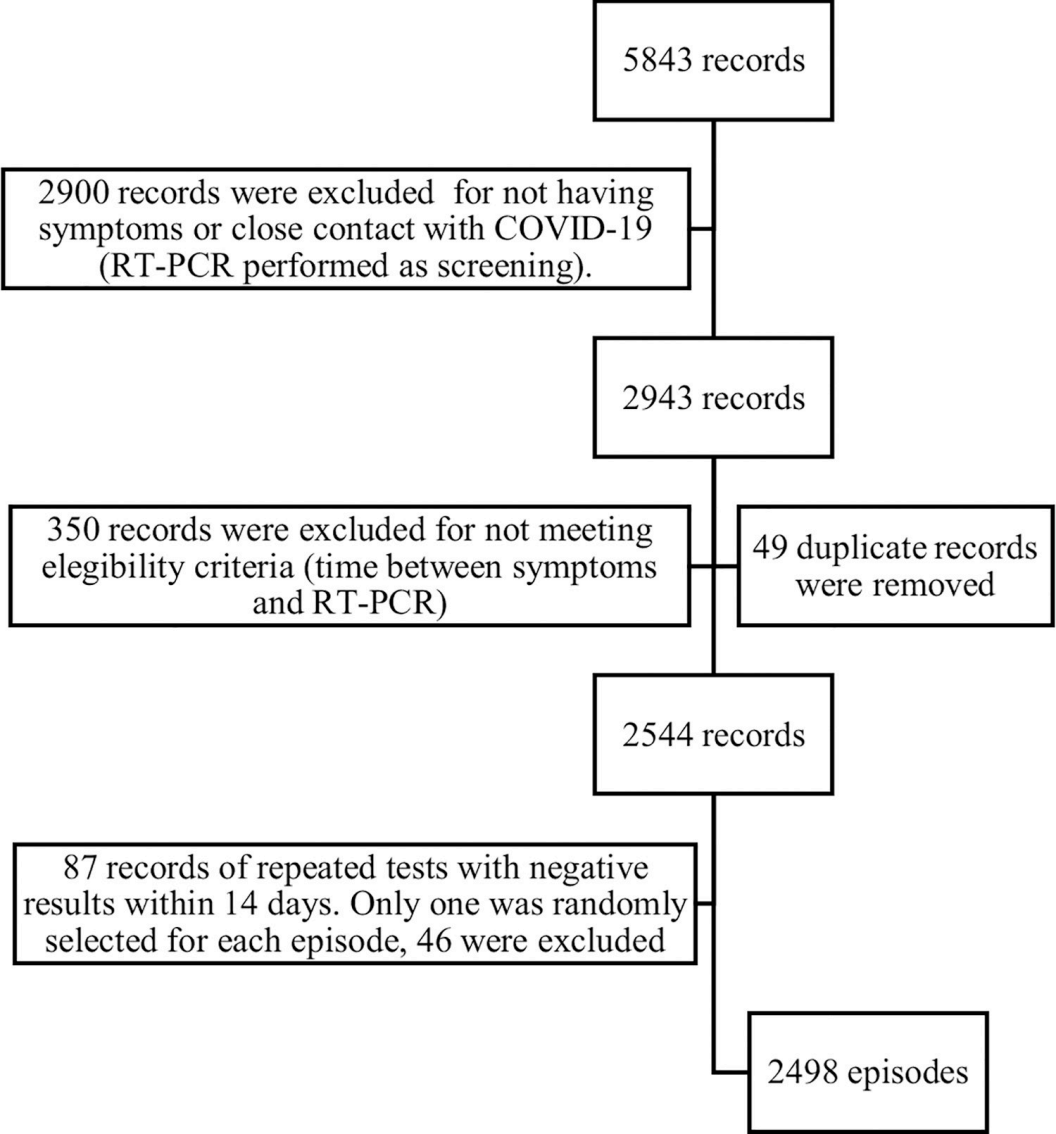
Episodes of suspected SARS-CoV-2 infection among HCWs of Hospital Universitario San Ignacio from March 6, 2020 to February 2, 2022, selected for the study according to eligibility criteria.

After assessing 5,483 episodes from Linea Respira, we applied eligibility criteria selecting 2,498 episodes of suspected SARS-CoV-2 infection (Fig 1). The episodes occurred in 1,733 HCWs, from whom 68.7% (n: 1,191) experienced a single episode, 21.8% (n: 378) had two episodes, 7.2% (n: 125) three episodes, and 2.3% (n: 39) had four or more episodes. The RT-PCR for SARS-CoV-2 was positive in 25% of the episodes (634 / 2,498).

The population’s median age was 32.3 years, with an interquartile range (IQR) of 27.3 to 33.9 years. Most of the episodes were in HCWs under 35 years of age (n: 1541, 61.7%) and in females (n: 1896, 75.9%). The episodes were more frequent in physicians (n: 656, 26.3%) and nurse assistants (n: 514, 20.6%).

The most frequently reported symptoms included odynophagia in 65.7% (n: 1,641), headache in 52.4% (n: 1,310), cough in 51.4% (n: 1,283), and fatigue/tiredness in 46.7% (n: 1,165) of cases. In 21.5% (n: 538) of the episodes the HCW had a history of previous SARS-CoV-2 infection, while in 52.6% (n: 1,313) of the HCW had completed the vaccination schedule. Table 1 outlines the characteristics of the episodes and the percentage with a positive RT-PCR result according to sociodemographic, potential occupational and non-occupational exposure to SARS-CoV-2, clinical and local epidemiological characteristics.

**Table 1:** Characteristics of the episodes of suspected infection with SARS-CoV-2 and percentage with a positive RT-PCR result in HUSI healthcare workers, from March 6, 2020 to February 2, 2022.

Characteristics	Episodes (N = 2498)*	Percentage of the episodes with an RT-PCR positive for SARS-CoV-2^†^
**Age (years), (n, %)**		
< 35	1541 (61.7%)	374 (24.3%)
35–44	663 (26.5%)	179 (27.0%)
≥ 45	294 (11.8%)	81 (27.6%)
**Sex at birth (n, %)**		
Female	1896 (75.9%)	484 (25.5%)
Male	602 (24.1%)	150 (24.9%)
**Socioeconomic status (n, %)**		
Low	829 (33.2%)	222 (26.8%)
Middle	1317 (52.8%)	348 (26.4%)
High	352 (14%)	64 (18.2%)
**Type of occupation (n, %)**		
Physician	656 (26.3%)	137 (20.9%)
Nurse	382 (15.3%)	96 (25.1%)
Nurse assistant	514 (20.6%)	154 (30.0%)
Administrative	487 (19.5%)	137 (28.1%)
Other	459 (18.4%)	110 (24.0%)
**Main service of the HCW (n, %)**		
Emergency room	386 (15.5%)	88 (22.8%)
General wards	820 (32.8%)	225 (27.4%)
Intensive care unit	163 (6.5%)	52 (31.9%)
Surgical areas	287 (11.5%)	62 (21.6%)
Ambulatory and diagnostic services	479 (19.2%)	125 (26.1%)
Administrative offices and other workers not in direct contact with patients.	363 (14.5%)	82 (22.6%)
**Shift (n: 2394), (n, %)**		
Day shift	1600 (66.8%)	415 (25.9%)
Night shift	794 (33.2%)	197 (24.8%)
**COVID-19 work area (n: 2449), (n, %)**		
Yes	1202 (49.1%)	305 (25.4%)
No	1247 (50.9%)	316 (25.3%)
**Close contact (n, %)** ^‡^		
Yes	633 (25.3%)	159 (25.1%)
No	1865 (74.7%)	475 (25.5%)
**Underlying medical conditions (n, %)** ^§^		
Cardiovascular and metabolic disease	299 (12.0%)	74 (24.7%)
Obesity	162 (6.5%)	50 (30.9%)
Asthma	163 (6.5%)	30 (18.4%)
Others^‖^	216 (8.6%)	63 (29.2%)
**Symptoms**^¶^ **(n, %)**		
Fever or chills	337 (13.4%)	131 (38.9%)
Cough	1283 (51.4%)	398 (31.0%)
Dyspnea	109 (4.4%)	38 (34.9%)
Rhinorrhea	1128 (45.2%)	269 (23.8%)
Odynophagia	1641 (65.7%)	423 (25.8%)
Anosmia and dysgeusia	236 (9.4%)	132 (55.9%)
Headache	1310 (52.4%)	319 (24.4%)
Fatigue/weakness	1165 (46.7%)	342 (29.4%)
Diarrhea	256 (10.2%)	51 (19.9%)
Myalgia or arthralgia	19 (0.8%)	10 (52.6%)
**History of SARS-CoV-2 infection (n, %)** **	538 (21.5%)	76 (14.1%)
**SARS-CoV-2 vaccination schedule (n, %)** ^††^		
No	1060 (42.4%)	338 (31.9%)
Incomplete schedule	125 (5.0%)	17 (13.6%)
Complete schedule	1313 (52.6%)	279 (21.2%)
**SARS- CoV-2 tests positivity (%) in Bogotá at the time of taking the test**^‡‡^ **(median, IQR)**	28.9 (16.8–36.4)	-
**Epidemic waves (n, %)** ^§§^		
First epidemic wave	348 (13.9%)	141 (40.5%)
Second epidemic wave	554 (22.2%)	168 (30.3%)
Third epidemic wave	919 (36.8%)	88 (9.6%)
Fourth epidemic wave	677 (27.1%)	237 (35.0%)

*: Column-based percentages

**†:** Row-based percentages. The denominators are those in the corresponding categories of the preceding column

**‡**: HCWs who were less than 6 feet away from a SARS-CoV-2-infected person (laboratory-confirmed or a clinical diagnosis) for 15 min without PPE, at any time since March 2020

**§:** Self-reported pre-existing medical condition

**‖:** Others underlying conditions: Anemia, history of tuberculosis, venous thromboembolic disease, osteoarthritis, migraine, epilepsy, HIV, kidney disease and rheumatological disease, neoplasia, and others

¶: Symptoms are not mutually exclusive, consequently, the percentages do not add to 100%

**: History of SARS-CoV-2 infection confirmed by RT-PCR, antibody, or antigen

**††:** An incomplete vaccination schedule was defined as only having one dose of the vaccine. Johnson and Johnson vaccine was not used as standard strategy in HUSI. A complete vaccination schedule was defined as having the complete basic schedule with or without a booster dose. Johnson and Johnson vaccine was not used as standard strategy in HUSI

**‡‡**: Percentage of SARS-CoV-2 tests positivity in the city of Bogotá on the day before the healthcare worker had the RT-PCR test done

**§§**: The episodes were assigned to the first, second, third and fourth epidemic waves according to the following dates: March 6, 2020, to September 30, 2020; October 1, 2020, to February 28, 2021; March 1, 2021, to October 30, 2021; and from November 1, 2021, to February 2, 2022, respectively.

### Selected prediction model

**Fig 2:**
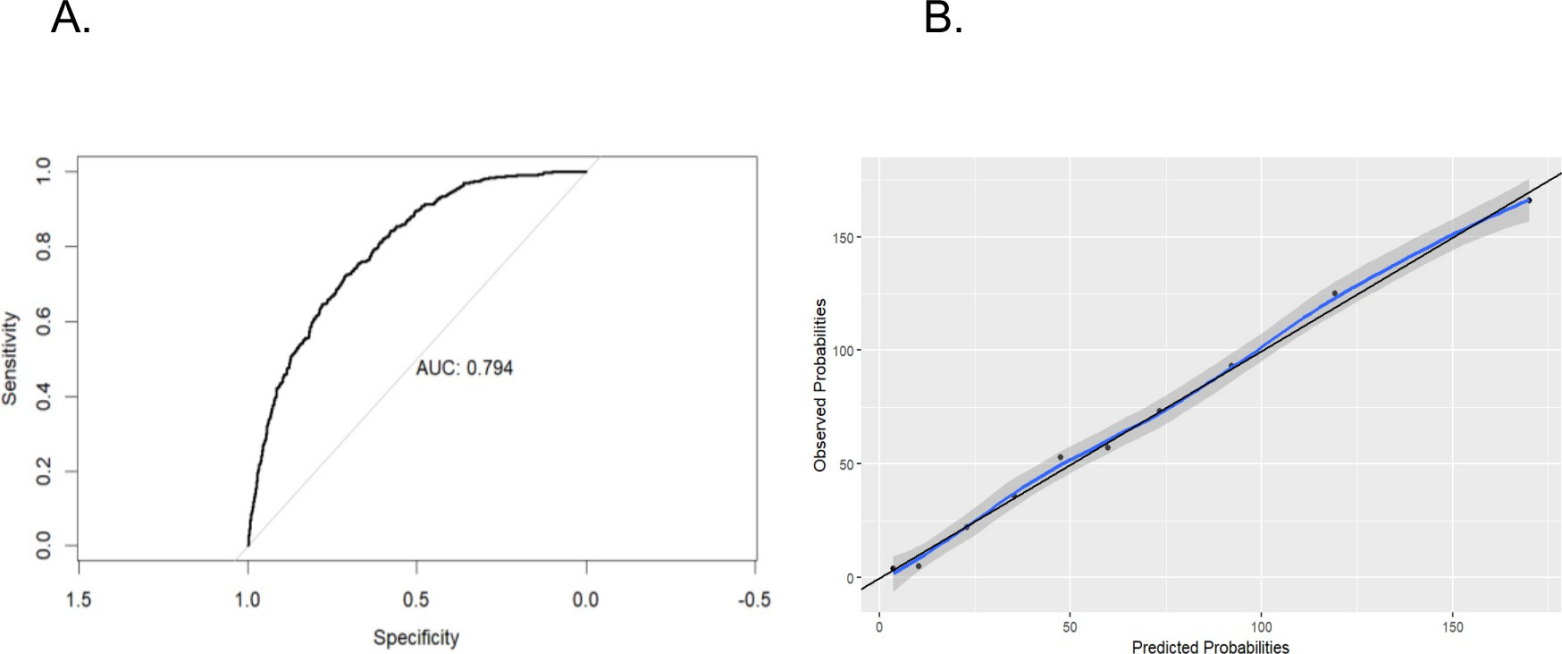
“Diagnostic performance of the selected model for the prediction of a positive RT-PCR result for SARS-CoV-2 in healthcare workers with suspected infection in a hospital setting. A. Receiver Operating Characteristic (ROC) curve. B. Calibration graph of the model”.

The selected prediction model is presented in Table 2. It achieved an AUC of 0.79, with a 95% confidence interval (CI) of 0.77 to 0.81 (Fig 2).

**Table 2:** Model selected for the prediction of a positive RT-PCR result for SARS-CoV-2 in healthcare workers with suspected infection in a hospital setting.

Predictors	*Βeta (Log-Odds)*	*95% CI* *	*Adjusted OR*	*95% CI* *
**(Intercept)**	-8.37	-10.10 – -6.68		
**Age (log)**	0.55	0.13 – 0.98	1.74	1.14–2.66
**Socioeconomic status**				
Low	ref.			
Middle	0.16	-0.09 – 0.41	1.17	0.92–1.51
High	-0.24	-0.69 – 0.20	0.78	0.50–1.22
**Occupation**				
Administrative	ref.			
Physician	-0.48	-0.88 – -0.07	0.62	0.41–0.93
Nurse	-0.46	-0.86 – -0.07	0.63	0.42–0.93
Nurse assistant	-0.13	-0.51 – 0.24	0.87	0.60–1.28
Other	-0.48	-0.85 – -0.11	0.62	0.43–0.90
**Main service**				
Administrative office	ref.			
Emergency room	0.47	0.03 – 0.91	1.60	1.03–2.48
General wards	0.71	0.30 – 1.12	2.03	1.35–3.07
Intensive care unit	0.71	0.18 – 1.24	2.03	1.20–3.44
Surgery areas	0.29	-0.20 – 0.78	1.34	0.82–2.18
Ambulatory and diagnostic services	0.43	0.03 – 0.84	1.54	1.03–2.31
**Symptoms**				
Fever and chills	0.76	0.45 – 1.07	2.14	1.57–2.90
Cough	0.7	0.48 – 0.92	2.01	1.62–2.50
Fatigue/weakness	0.25	0.04 – 0.46	1.29	1.04–1.59
Diarrhea	-0.58	-0.96 – -0.22	0.56	0.38–0.80
Anosmia or dysgeusia	1.6	1.25 – 1.96	4.97	3.50–7.11
**Asthma**	-0.46	-0.94 – -0.02	0.63	0.39–0.98
**History of SARS-CoV-2 infection** ^†^	-0.98	-1.30 – -0.68	0.37	0.27–0.51
**SARS-CoV-2 Vaccination** ^‡^				
Not vaccinated	ref.			
Incomplete schedule	-0.65	-1.26 – -0.08	0.52	0.28–0.92
Complete schedule	-0.39	-0.61 – -0.16	0.68	0.54–0.85
**SARS- CoV-2 tests positivity (%)**^§^ **(logarithm)**	1.48	1.25 – 1.73	4.4	3.50–5.63
**Interaction: Fever and chills and anosmia or dysgeusia**	-1.03	-1.82 – -0.22	0.36	0.16–0.80

This model has an Akaike information criterion (AIC) of 2320, deviance of 2272 and Log-Likelihood of -1136

*: CI: Confidence interval

**†:** History of SARS-CoV-2 infection confirmed by RT-PCR, antibody or antigen

**‡:** An incomplete vaccination schedule was defined as having only one dose of the vaccine. Johnson and Johnson vaccine was not used as standard strategy in HUSI. A complete vaccination schedule was defined as having the complete schedule with or without a booster dose. Johnson and Johnson vaccine was not used as standard strategy in HUSI

**§:** Percentage of SARS-CoV-2 tests positive in the city of Bogotá on the day before the healthcare worker had the RT-PCR test done.

To determine the cut-off point, the Youden method was initially employed to balance sensitivity and specificity. However, this analysis resulted in a sensitivity of 72% and a specificity of 71.1%, which may limit its applicability for clinical decision-making. Consequently, specificity was prioritized, leading to the selection of a cut-off point of 0.49, achieving a specificity of 93% and a sensitivity of 36%, with a positive likelihood ratio (LR+) of 5.3 and a negative likelihood ratio (LR–) of 1.5. The model demonstrated adequate calibration, confirmed both graphically and through the Hosmer-Lemeshow test, which yielded a p-value of 0.785 (Fig 2).

Additionally, a CORP approach was employed, displaying the recalibrated forecast probabilities in a reliability diagram, also documenting an adequate calibration by this methodology (miscalibration of 0.002) (S1 Fig).

Considering that the use of the selected model requires up-to-date and exact knowledge of the positivity of tests for SARS-CoV-2 in the relevant population, we developed an additional model in which the local tests positivity variable is categorical instead of continuous. It has two categories representing low (less than 15% tests positivity) or high circulation of SARS-CoV-2 (15% or higher positivity) in the population. This approach would facilitate the use of the model when the exact population tests positivity is unknown (S1 Table). This modified model showed an AUC of 0.77 with a 95% IC of 0.76–0.80. An adequate calibration of the model was observed graphically, and with the Hosmer and Lemeshow test a p-value of 0.063 was obtained (S2 and S3 Figs).

### Internal validation

The internal validation of the selected model was carried out through a 10-fold cross validation technique, finding an average AUC of 0.78 (95% CI 0.77–0.78) (S2 Table). Additionally, in the temporal validation that was carried out using exclusively the episodes that occurred when Omicron variant was dominant in the fourth epidemic wave, an AUC of 0.81 (95% CI 0.78–0.84) was found for the model, with adequate calibration (Hosmer and Lemeshow test, p-value = 0.37) (S4 Fig).

### Validation of the prediction model in periods with low levels of SARS-CoV-2 circulation

**Fig 3:**
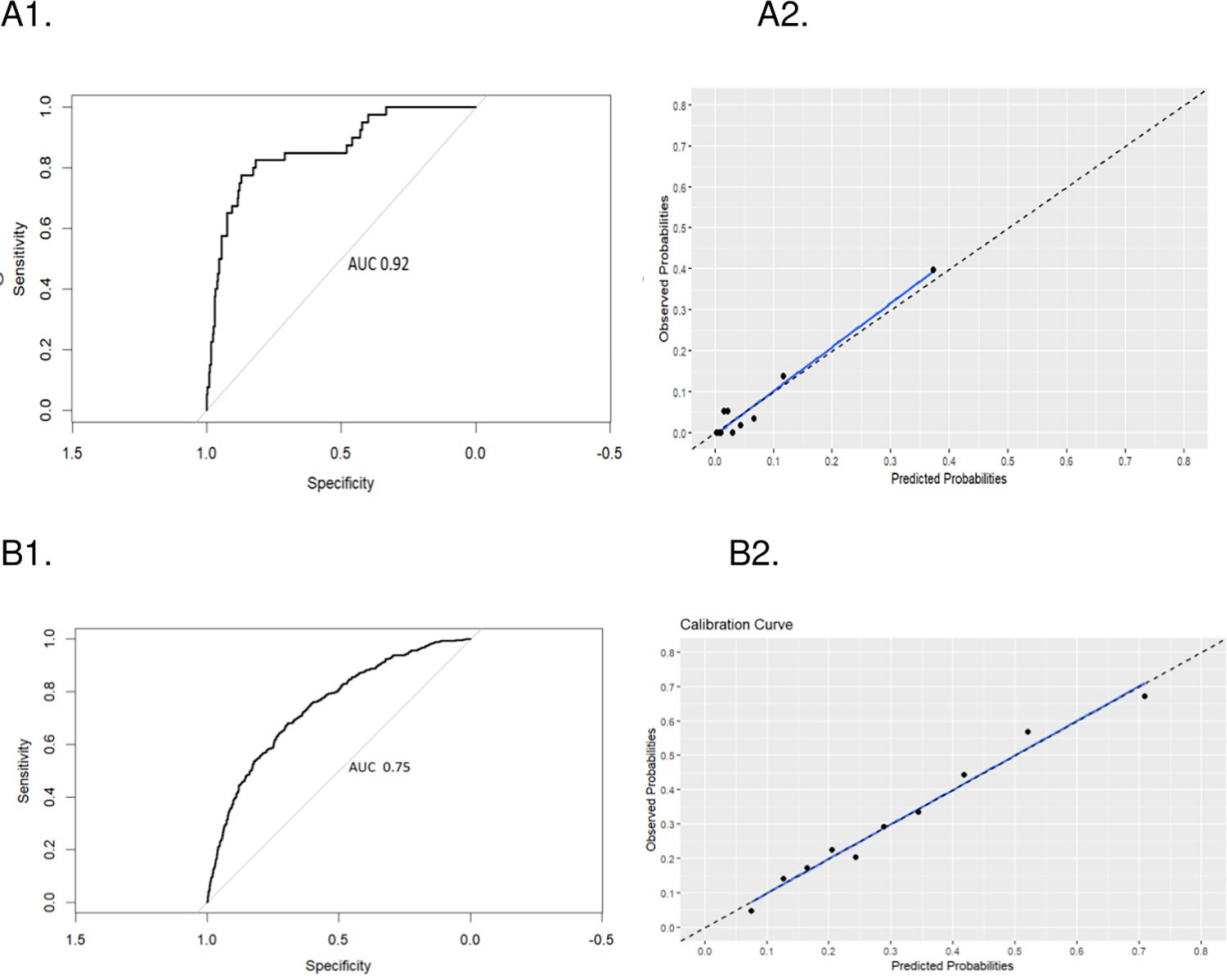
Diagnostic performance of the selected model for the prediction of a positive RT-PCR result for SARS-CoV-2 in healthcare workers with suspected infection in a hospital setting under conditions of low (RT-PCR positivity in the city below 15%) and high circulation (RT-PCR positivity in the city 15% or higher) of the virus. **A1.** Receiver Operating Characteristic (ROC) curve and **A2.** Calibration graph for the prediction model during periods when the population RT-PCR positivity is below 15% (low positivity). **B1.** Receiver Operating Characteristic (ROC) curve and **B2.** Calibration graph for the prediction model during periods when the population RT-PCR positivity is 15% or higher (high positivity).

In the validation of the model on 580 episodes that occurred when SARS-CoV-2 RT-PCR positivity in Bogotá was less than 15%, an AUC of 0.93 (95% CI 0.90–0.95) was found, achieving adequate calibration (Hosmer and Lemeshow test, p-value = 0.12) (Fig 3). The sensitivity and specificity of the model with a cut-off point at 0.49 were 40% and 97%, respectively. For instance, if the positivity were 10%, this model would correctly classify 91% of HCWs, with only 6% false negatives and 2.7% false positives (S3 Table).

### Validation of the prediction model in periods with high levels of SARS-CoV-2 circulation

In the validation of the model on 1918 episodes that occurred when the SARS-CoV-2 RT-PCR positivity in Bogotá was equal or greater than 15%, an AUC of 0.75 (95% CI 0.72–0.77) was found, achieving adequate calibration (Hosmer and Lemeshow test, p-value 0.41) (Fig 3). The sensitivity and specificity of the model with a cut-off point at 0.49 were 30% and 93%, respectively. For instance, if the positivity were 30%, this model would correctly classify 74% of HCWs, with 5% false positives and 21% false negatives (S3 Table).

## Discussion

Despite the ending of the COVID-19 pandemic emergency declared by WHO in May 2023, timely diagnosis of SARS-CoV-2 infections remains an important challenge for HCWs. It is essential to continue prioritizing suspected cases among healthcare workers (HCWs) to ensure timely access to diagnostic testing. Our model predicts RT-PCR positive results for SARS-CoV-2 among HCWs with suspected infection with a global accuracy of AUC 0.79 (95% CI 0.77–0.81) and a specificity of 93% at the defined cut-off point.

One crucial aspect of this model is the inclusion of a variable to capture the local and temporal variations in SARS-CoV-2 epidemiological situation. This factor stands as a key element in predicting the risk of infection and significantly enhances the model’s performance. We identified only one model developed in the general population that assessed diagnostic performance based on different RT-PCR positivity scenarios. However, this model did not include this variable among its predictors [27].

Based on our model’s performance, we suggest using it differently based on the epidemiological situation. In low SARS-CoV-2 circulation periods, the model could serve as a replacement diagnostic test to classify HCWs as infected or not, potentially reducing the need for RT-PCR. Conversely, in high viral circulation periods, the model could be used as a triage test due to its high specificity. If the model indicates a high probability of a positive RT-PCR result, HCWs should be considered potentially infected and advised to start isolation protocols. If the model indicates a low probability of a positive RT-PCR result, HCWs should undergo a molecular COVID-19 test to reduce false negatives, as the model has shown limited sensitivity. In resource-limited settings, this model can help prioritize testing and reduce unnecessary expenses.

Moreover, our model has the advantage that it was developed using data from multiple epidemic waves, including the period when the Omicron variant, currently prevailing, was predominant and many HCWs had already received the COVID-19 vaccine. During the predominance of the Omicron variant, symptom patterns varied, with a reduced frequency of anosmia/dysgeusia and an increased incidence of upper respiratory symptoms. [28–30]. When we conducted internal validation solely on data from Omicron-dominant fourth wave, our model demonstrated satisfactory discrimination and calibration. This finding is relevant, as it suggests that our model may still be applicable now when Omicron subvariants, including JN.1 and its descendants are prevalent, as reports indicate a similar symptom pattern across these subvariants [31,32].

Other important variables incorporated into our model were those associated with the HCWs occupation. Remarkably, these variables have not been included in previously reported models for this specific population in the reviewed literature [18,33]. However, previous studies have identified occupation-related factors like occupation type, and the specific healthcare service in which workers are employed, as significant risk factors for SARS-CoV-2 [5–7].

Regarding symptom inclusion in our prediction model, we have integrated common symptoms observed in SARS-CoV-2 infected patients, including anosmia/dysgeusia, fever, cough, and fatigue. We have also considered the presence of diarrhea, which may decrease the probability of infection [34]. It is important to note that relying solely on symptoms for prediction may not yield strong diagnostic performance [34,35].

In contrast to previous models developed during the pandemic, the prediction model in this study does not rely on a variety of diagnostic tests, such as biomarkers or chest x-rays [17,36]. This is advantageous as most SARS-CoV-2 infections in HCWs are currently mild, and only clinical and occupational information is typically needed, eliminating the need for extra testing [27,37]. This model is recommended for use in HCWs with suspected mild COVID-19, but not for severe cases requiring hospitalization. In such cases, RT-PCR tests are recommended for etiological diagnosis and treatment decisions.

Our study has limitations. First, while it involves HCWs with suspected SARS-CoV-2 infection during the Omicron variant’s predominance, it was not evaluated in episodes occurring when other prominent Omicron sublineages (XBB.2.1, EG. 5.1 or JN.1) were circulating in 2023 and 2024 [38]. This highlights the importance of future external and temporal validation of the model in these specific and evolving epidemiological scenarios, especially considering the circulation of other respiratory viruses.

Secondly, our model does not include factors like HCWs’ use of personal protective equipment, as accurately measuring these variables is difficult. HCWs tend to over-report compliance when monitored in their workplace [39]. Thirdly, in low viral circulation scenarios, this model can be used as a substitute for RT-PCR in HCWs with mild symptoms. In a scenario with a 10% prevalence, 9% of workers may be misclassified: 3% as false positives, potentially leading to unnecessary isolation, and 6% as false negative results, allowing infected individuals to continue working. In these instances, it is imperative for HCWs with respiratory symptoms to wear masks and practice frequent hand washing to reduce the risk of transmitting SARS-CoV-2 to patients.

To properly implement this model, physicians guided by local occupational health services or infection control departments should apply it. They must know the local COVID-19 epidemiology and input local data on positive SARS-CoV-2 tests positivity and HCWs’ individual variables in the model. If exact values for SARS-CoV-2 tests positivity are unavailable, the model with the dichotomous variable may be employed instead, only requiring knowledge of high or low SARS-CoV-2 circulation.

### Suggested use of the predictive model

To improve readers’ understanding of the predictive model and facilitate its application for predicting SARS-CoV-2 infection among HCWs in similar settings, we developed a calculator based on it (https://github.com/Mars1971/SARS_CoV_2_Risk_Prediction_in_Healthcare_Workers/blob/main/README.md). This tool is licensed under the Creative Commons Attribution-NonCommercial-ShareAlike 4.0 International license CC BY-NC-SA 4.0.

To use the calculator, users will input data for the following variables of the HCW that constitute the prediction model: age in years, socioeconomic status (low, middle and high according to local definitions. For instance, in our country, it depends on the economic strata that are used to classify the place of residency), occupation, main hospital service where the person works, symptoms at presentation (fever and chills, cough, fatigue/weakness, diarrhea, anosmia or dysgeusia), history of asthma, history of SARS-CoV-2 infection, SARS-CoV-2 vaccination status (not vaccinated, incomplete schedule, complete schedule with or without booster).

It is also essential for the prediction to input an estimate of the local level of viral circulation when the HCW presents for care. An approximation to this level is based on the positivity of the SARS-CoV-2 tests in the population. We recommend that users obtain this positivity percentage from local public health authorities or their institution’s occupational health team.

Based on the results, the calculator also provides guidance on the need or not for additional testing. In the context of a respiratory peak with high levels of SARS-CoV-2 circulation in the population (test positivity equal or greater than 15%) and prediction of a high probability of infection, the healthcare worker should be managed as potentially infected, and isolation protocols should be initiated without further testing. Conversely, if the calculator estimates a low probability of infection for the HCW, a molecular COVID-19 test is recommended to reduce the risk of false negatives, as the model has demonstrated limited sensitivity and the risk of being infected is relatively high given the epidemiological situation.

In the context of low to moderate levels of SARS-CoV-2 circulation in the population (test positivity below 15%) and a prediction of a high probability of infection, we suggest that the healthcare worker be considered potentially infected, without further testing. However, if the model predicts a low probability of infection in this context, the worker can be considered not infected.

## Conclusion

In conclusion, we present an innovative diagnostic prediction model for a positive RT-PCR result for SARS-CoV-2 that among its predictors includes an indicator of the local epidemiological situation and may serve as a decision-making tool that help to spare tests and save resources in programs for COVID-19 control in HCW, particularly in resource-limited settings, where access to diagnostic tests is expensive, lacking, or limited. This model could be utilized differentially based on the epidemiological situation of SARS-CoV-2, either as a replacement for the reference test in periods of low viral circulation, or as a triage test in high-circulation periods.

## Supporting information

S1 Fig Graphic of the recalibrated forecast probabilities of model selected for the prediction of a positive RT-PCR result for SARS-CoV-2 in healthcare workers with suspected infection in a hospital setting. CORP (consistency, optimization, reproducibility, and group-adjacent violator (PAV)-based algorithm) approach.
(DOCX)

S2 Fig A. Receiver operating characteristic (ROC) curve of the prediction model of a positive RT-PCR result for SARS-CoV-2 in healthcare workers with suspected infection in a hospital setting with the population SARS-CoV-2 tests positivity variable categorized. B. Calibration graph of the prediction model of a positive RT-PCR result for SARS-CoV-2 in healthcare workers with suspected infection in a hospital setting with the population SARS-CoV-2 tests positivity variable categorized.
(DOCX)

S3 Fig Graphic of the recalibrated forecast probabilities of the prediction model of a positive RT-PCR result for SARS-CoV-2 in healthcare workers with suspected infection in a hospital setting with the population SARS-CoV-2 tests positivity variable categorized. CORP (consistency, optimization, reproducibility, and group-adjacent violator (PAV)-based algorithm) approach.
(DOCX)

S4 Fig Internal validation of the model in the fourth epidemic wave (Omicron predominance). A. Receiver operating characteristic (ROC) curve of the internal validation of the model for the prediction of a positive RT-PCR result for SARS-CoV-2 in healthcare workers with suspected infection in a hospital setting in the fourth epidemic wave. B. Calibration graph of the model of the internal validation of the model for the prediction of a positive RT-PCR result for SARS-CoV-2 in healthcare workers with suspected infection in a hospital setting in the fourth epidemic wave.
(DOCX)

S1 Table Model for the prediction of a positive RT-PCR result for SARS-CoV-2 in healthcare workers with suspected infection in a hospital setting with the population SARS-CoV-2 tests positivity variable categorized. (DOCX)

S2 Table Cross-validation of model selected for the prediction of a positive RT-PCR result for SARS-CoV-2 in healthcare workers with suspected infection in a hospital setting. (DOCX)

S3 Table Diagnostic performance in periods with low and high level of SARS-CoV-2 circulation of the model selected for the prediction of a positive RT-PCR result for SARS-CoV-2 in healthcare workers with suspected infection in a hospital setting. (DOCX)

S1 Dataset Excel file with dataset used for the analysis. (XLSX)
