# Supplementary material for: Development of a diagnostic multivariable prediction model of a positive SARS-CoV-2 RT-PCR result in healthcare workers with suspected SARS-CoV-2 infection in hospital settings
Source: PLoS One. 2024 Dec 26;19(12):e0316207. doi: 10.1371/journal.pone.0316207 (PMC11670996; doi:10.1371/journal.pone.0316207)
Supplement: S1 Fig — CORP (consistency, optimization, reproducibility, and group-adjacent violator (PAV)-based algorithm) approach. (DOCX) [file pone.0316207.s001.docx]

**
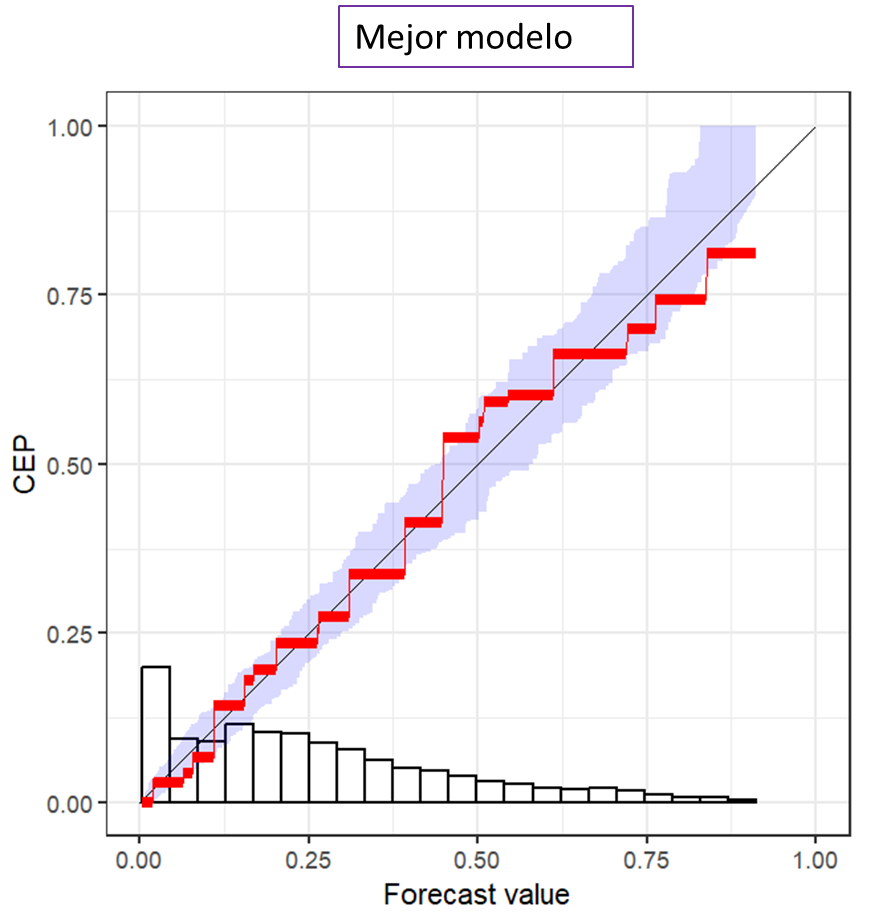
**

**S1 Figure.** Graphic of the recalibrated forecast probabilities of model selected for the prediction of a positive RT-PCR result for SARS-CoV-2 in healthcare workers with suspected infection in a hospital setting. CORP (consistency, optimization, reproducibility, and group-adjacent violator (PAV)-based algorithm) approach.
