# Supplementary material for: Development of a diagnostic multivariable prediction model of a positive SARS-CoV-2 RT-PCR result in healthcare workers with suspected SARS-CoV-2 infection in hospital settings
Source: PLoS One. 2024 Dec 26;19(12):e0316207. doi: 10.1371/journal.pone.0316207 (PMC11670996; doi:10.1371/journal.pone.0316207)
Supplement: S2 Fig — A. Receiver operating characteristic (ROC) curve of the prediction model of a positive RT-PCR result for SARS-CoV-2 in healthcare workers with suspected infection in a hospital setting with the population SARS-CoV-2 tests positivity variable categorized. B. Calibration graph of the prediction model of a positive RT-PCR result for SARS-CoV-2 in healthcare workers with suspected infection in a hospital setting with the population SARS-CoV-2 tests positivity variable categorized. (DOCX) [file pone.0316207.s002.docx]

1. B.


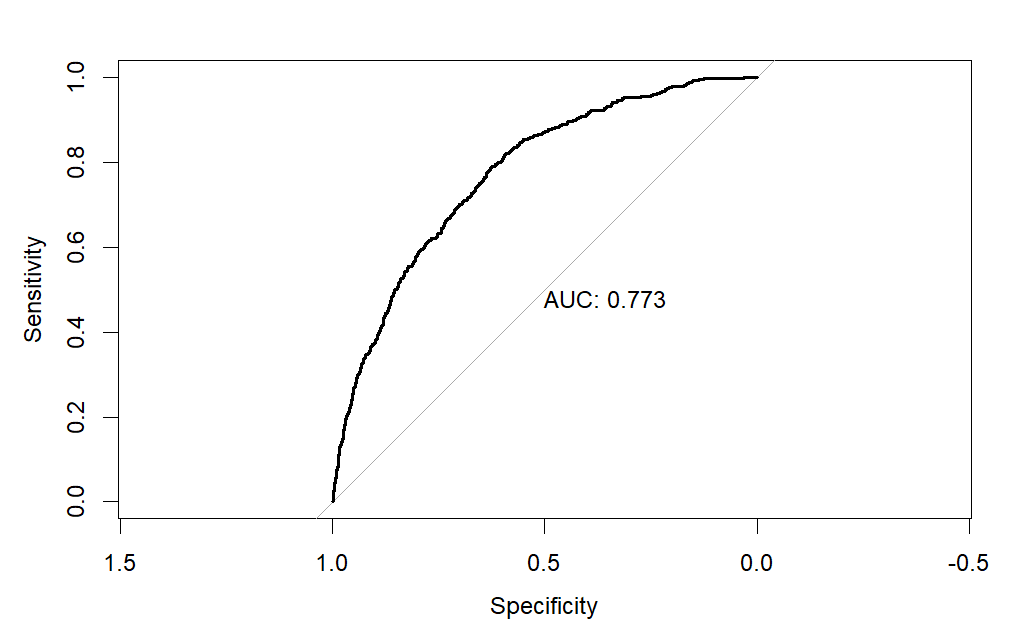

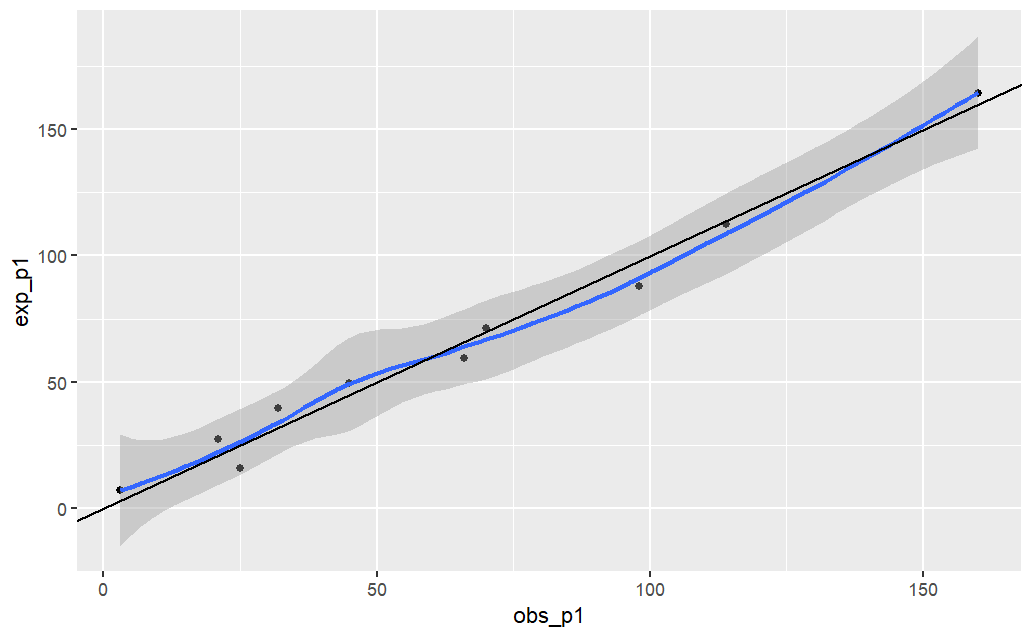


**S2 Figure.** A. Receiver operating characteristic (ROC) curve of the prediction model of a positive RT-PCR result for SARS-CoV-2 in healthcare workers with suspected infection in a hospital setting with the population SARS-CoV-2 tests positivity variable categorized. B. Calibration graph of the prediction model of a positive RT-PCR result for SARS-CoV-2 in healthcare workers with suspected infection in a hospital setting with the population SARS-CoV-2 tests positivity variable categorized.
