# Supplementary material for: Development of a diagnostic multivariable prediction model of a positive SARS-CoV-2 RT-PCR result in healthcare workers with suspected SARS-CoV-2 infection in hospital settings
Source: PLoS One. 2024 Dec 26;19(12):e0316207. doi: 10.1371/journal.pone.0316207 (PMC11670996; doi:10.1371/journal.pone.0316207)
Supplement: S1 Table — (DOCX) [file pone.0316207.s005.docx]

**S1 Table. Model for the prediction of a positive RT-PCR result for SARS-CoV-2 in healthcare workers with suspected infection in a hospital setting with the population SARS-CoV-2 tests positivity variable categorized.**

| ***Predictors*** | **Model with the population tests positivity variable categorized** | |
| --- | --- | --- |
|  | ***Log-Odds*** | ***95% CI^a^*** |
| **(Intercept)** | -5.18 | -6.70 – -3.67 |
| **Age (logarithm)** | 0.55 | 0.14 – 0.97 |
| **Socioeconomic status** |  |  |
| Low | ref. |  |
| Middle | 0.12 | -0.12 – 0.37 |
| High | -0.28 | -0.72 – 0.15 |
| **Occupation** |  |  |
| Administrative | ref. |  |
| Physician | -0.49 | -0.89 – -0.09 |
| Nurse | -0.47 | -0.86 – -0.09 |
| Nurse assistant | -0.17 | -0.54 – 0.19 |
| Other | -0.49 | -0.85 – -0.13 |
| **Main service** |  |  |
| Administrative office | ref. |  |
| Emergency room | 0.44 | 0.01 – 0.87 |
| General wards | 0.71 | 0.31 – 1.11 |
| Intensive care unit | 0.68 | 0.17 – 1.20 |
| Surgery areas | 0.24 | -0.24 – 0.72 |
| Ambulatory and diagnostic services | 0.39 | -0.00 – 0.78 |
| **Symptoms** |  |  |
| Fever and chills | 0.80 | 0.50 – 1.10 |
| Cough | 0.71 | 0.50 – 0.93 |
| Fatigue/weakness | 0.26 | 0.05 – 0.46 |
| Diarrhea | -0.57 | -0.94 – -0.22 |
| Anosmia or dysgeusia | 1.59 | 1.25 – 1.94 |
| **Asthma** | -0.46 | -0.92 – -0.02 |
| **History of SARS-CoV-2 infection^b^** | -0.92 | -1.23 – -0.63 |
| **SARS-CoV-2 Vaccination schedule^c^** |  |  |
| Not vaccinated | ref. |  |
| Incomplete schedule | -0.75 | -1.36 – -0.20 |
| Complete schedule | -0.14 | -0.37 – 0.08 |
| **Percentage of positivity of the RT-PCR in Bogotá^d^** |  |  |
| < 15% | ref. |  |
| ≥ 15% | 1.82 | 1.48 – 2.19 |
| **Interaction: Fever and chills and anosmia or dysgeusia** | -1.01 | -1.80 – -0.21 |

a.CI95%: 95% confidence Interval. b. History of SARS-CoV-2 infection confirmed by RT-PCR, antibody or antigen. c. An incomplete vaccination schedule was defined as a health worker who at the time of performing the RT-PCR only had one dose of the biological agent. A complete vaccination schedule was defined as the health worker who, at the time of performing the RT-PCR had the complete biological schedule with or without booster. d. Categorized percentage of positivity in the city of Bogotá on the day before the health worker performed the RT-PCR test for SARS-CoV-2.
