# Supplementary material for: Development of a diagnostic multivariable prediction model of a positive SARS-CoV-2 RT-PCR result in healthcare workers with suspected SARS-CoV-2 infection in hospital settings
Source: PLoS One. 2024 Dec 26;19(12):e0316207. doi: 10.1371/journal.pone.0316207 (PMC11670996; doi:10.1371/journal.pone.0316207)
Supplement: S2 Table — (DOCX) [file pone.0316207.s006.docx]

| **S2 Table. Cross-validation of model selected for the prediction of a positive RT-PCR result for SARS-CoV-2 in healthcare workers with suspected infection in a hospital setting.** | | | | | | | | | | | | | |
| --- | --- | --- | --- | --- | --- | --- | --- | --- | --- | --- | --- | --- | --- |
|  |  | **Subset 1** | **Subset 2** | **Subset 3** | **Subset 4** | **Subset 5** | **Subset 6** | **Subset 7** | **Subset 8** | **Subset 9** | **Subset 10** | **AUC Average**  **(CI 95%)** |  |
| Selected model | Discrimination | 0.78 | 0.77 | 0.78 | 0.78 | 0.78 | 0.78 | 0.78 | 0.77 | 0.78 | 0.78 | 0.78 (0.77-0.78) |  |
