# Supplementary material for: Development of a diagnostic multivariable prediction model of a positive SARS-CoV-2 RT-PCR result in healthcare workers with suspected SARS-CoV-2 infection in hospital settings
Source: PLoS One. 2024 Dec 26;19(12):e0316207. doi: 10.1371/journal.pone.0316207 (PMC11670996; doi:10.1371/journal.pone.0316207)
Supplement: S3 Table — (DOCX) [file pone.0316207.s007.docx]

|  | **Low level of SARS-CoV-2 circulation. (10%)^a^** | | **High level of SARS-CoV-2 circulation. (30%)^b^** | | | |  |
| --- | --- | --- | --- | --- | --- | --- | --- |
|  | **n** | **%** | **n** | | **%** | |  |
| **True positive** | 4000 | 4 | | 9000 | | 9 | |
| **False negative** | 6000 | 6 | | 21000 | | 21 | |
| **False positive** | 2700 | 2.7 | | 4900 | | 4.9 | |
| **True negative** | 87300 | 87.3 | | 65100 | | 65.1 | |

**S3 Table. Diagnostic performance in periods with low and high level of SARS-CoV-2 circulation of the model selected for the prediction of a positive RT-PCR result for SARS-CoV-2 in healthcare workers with suspected infection in a hospital setting.**

1. A calculation is performed considering a population of 100,000 workers, a sensitivity of 49%, a specificity of 97%, and a prevalence of 10%. The sensitivity and specificity values were obtained from the internal validation of the selected model when applying it to episodes that occurred when the positivity for SARS-CoV-2 tests in Bogotá was less than or equal to 15%.
2. A calculation is performed considering a sample of 100,000 workers, a sensitivity of 30%, a specificity of 93% and a prevalence of 30%. The sensitivity and specificity values were obtained from the internal validation of the selected model when applying it to episodes that occurred when the positivity for SARS-CoV-2 tests in Bogotá was greater than 15%.
